# Development of a global forearm reconstruction system for post-tumor resection defects of the radius or ulna: a proof-of-concept study

**DOI:** 10.3389/fbioe.2025.1547652

**Published:** 2025-06-11

**Authors:** Haijie Liang, Jie Zang, Siyi Huang, Boyang Wang, Shun Tang, Zhiye Du, Feiyang Qi, Wei Guo, Jichuan Wang, Xiaodong Tang

**Affiliations:** ^1^ Musculoskeletal Tumor Center, Peking University People’s Hospital, Beijing, China

**Keywords:** 3D printing, ulna, radius, endoprosthesis, tumor

## Abstract

**Background:**

Bone defects resulting from sarcoma resection in the forearm present significant challenges for reconstruction, with limited guidance available in the literature.

**Methods:**

We developed a novel series of 3D-printed endoprostheses, called the Global Forearm Reconstruction System (GFRS), to reconstruct defects of the proximal radius (PR), distal ulna (DU), total ulna (TU), and total radius (TR). Finite element analysis (FEA) was performed to determine the mechanical support function of the GFRS endoprostheses. We also tested the rotatory function of the endoprostheses *ex vivo* using a resin model. Finally, we summarized the preliminary outcomes of three pediatric cases using the GFRS endoprostheses for reconstruction.

**Results:**

Resection of PR, DU, TU and TR leads to stress concentration in the remaining structures, which can be mitigated by the corresponding GFRS endoprostheses. The novel endoprostheses demonstrated full supination capability and approximately 50% of pronation in the *ex vivo* model. All of the three clinical cases achieved satisfactory functional status (MSTS-93:28-29; MEPS: 95-100) without complications during mid-term follow-up (32–42 months).

**Conclusion:**

In this proof-of-concept study, we demonstrated that the GFRS endoprostheses not only meet the theoretical reconstruction requirements but also exhibit a good safety profile and produce satisfactory functional outcomes in a preliminary cohort with mid-term follow-up.

## 1 Introduction

The forearm is a complex anatomical structure composed of two bones,the radius and ulna,as well as four joints—the elbow, wrist, and proximal and distal ulnoradial joints (PURJ and DURJ. These components are stabilized by articular capsules, the intraosseous membrane, and supporting ligaments, and mobilized through synergic actions of various muscles. Together, these elements facilitate essential forearm functions, including: (1) providing mechanical support between the carpus and humerus, (2) maintaining stability and mobility of the elbow and wrist joints, and (3) enabling pronation and supination of the forearm (Kaufmann et al., 2002; Bu et al., 2006; Shaaban et al., 2006; Kleinman, 2007; Samora, 2021).

For patients with primary bone or soft tissue sarcomas of the forearm, *en bloc* resection is necessary to achieve local control of the disease. Such resections result in substantial bone defects that require meticulous reconstruction to restore the intricate functionality of the forearm. While previous studies have reported successful reconstructions for defects in the distal radius, proximal ulna, and intercalary regions of the radius and ulna (Liang et al., 2022a; Adani et al., 2004; Yu et al., 2014; Chobpenthai et al., 2020; Lunn et al., 2021), significant technical challenges persist for reconstructing defects involving the proximal radius (PR), distal ulna (DU), total ulna (TU), and total radius (TR).

The advancement of metal 3D-printing technology has expanded new possibilities to improve traditional reconstruction techniques and address unresolved challenges (Liang et al., 2022a; Liang et al., 2022b; Ji et al., 2020). For example, our prior research involving a 3D-printed proximal ulnar endoprosthesis with hemiarthroplasty demonstrated effectiveness in restoring elbow function while mitigating the risk of mechanical failure (Liang et al., 2022a). However, conventional reconstruction approaches have been proven inadequate for addressing all three essential functions for defects in the PR, DU, TU, or TR.

To address this challenge, we developed a series of endoprostheses, collectively referred to as the Global Forearm Reconstruction System (GFRS). This study aimed to investigate the following questions: (1) Could the GFRS endoprostheses improve stress distribution in residual bones, as indicated by finite element analysis? (2) Did the GFRS endoprostheses enable pronation and supination in a resin model? (3) What were the safety and efficacy outcomes of the GFRS endoprostheses in clinical applications?

## 2 Materials and methods

### 2.1 Design of the GFRS endoprostheses

**FIGURE 1:**
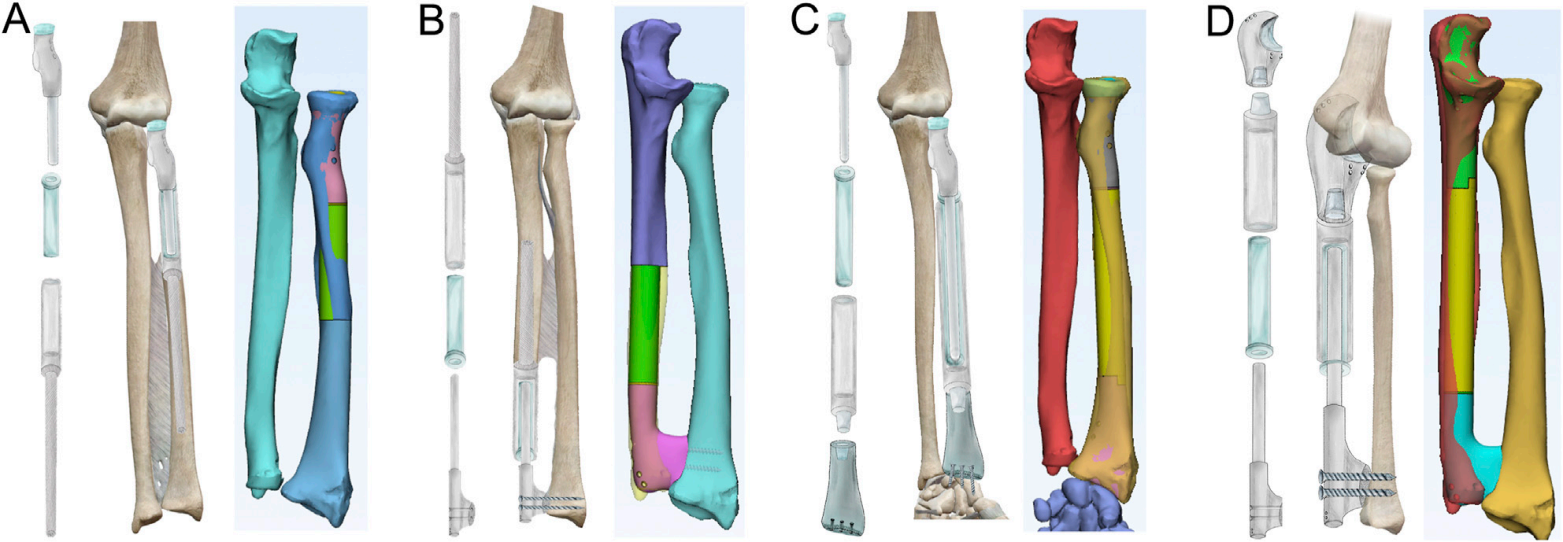
Designs of 3D-printed GFRS endoprostheses. **(A)** The proximal radial endoprosthesis consists of a proximal component with a smooth pole, a bushing, and a distal component with an intramedullary stem. Once assembled, the three parts form a telescoping structure that allows for rotation. The proximal component includes suture holes for annular ligament reconstruction. **(B)** The distal ulnar endoprosthesis features a proximal component with an intramedullary stem, a bushing, and a distal component with a smooth pole. The distal component has screw trajectories for fixation of the distal radioulnar joint. **(C)** The total radial endoprosthesis is designed with four components, similar to the telescoping structure of the other endoprostheses. The distal component is engineered for wrist arthrodesis, with predefined screw trajectories. **(D)** The total ulnar endoprosthesis also features four components with a similar telescoping structure. The proximal component is designed to enable hemiarthroplasty, as previously described.

The design of the GFRS endoprostheses was guided by the functional requirements of the forearm. Mechanical stability was ensured by reconstructing the dual-bone structure, while joint articulation was restored through soft-tissue reconstruction for the proximal joints (e.g., elbow, PURJ) and screw fixation for the distal joints (e.g., wrist, DURJ). A telescoping mechanism, comprising a smooth metal rod within a polyethylene bushing, was employed to facilitate forearm rotation (Figure 1).

The proximal components of the PR and TR endoprostheses were designed based on DICOM data derived from CT scans (Figures 1A,C). To prevent dislocation of the radial head while preserving humeroradial joint mobility, two or three suture holes were included to reattach the annular ligament. Fixation of the PR and DU endoprostheses was achieved using an uncemented intramedullary stem.

For the reconstruction of the DURJ in the DU and TU endoprostheses, we adapted the concept of APTIS prosthesis (APTIS Medical, Louisville, KY, United States) (Laurentin-Perez et al., 2008) by incorporating a distal fusion component (Figures 1B,D). Fusion of the DURJ was facilitated by a 3D-printed porous interface and initial fixation with two screws. For the TR endoprosthesis, radiocarpal arthrodesis was achieved using three compressive screws and a 3D-printed porous interface (Figure 1C). Finally, for the TU endoprosthesis, a previously reported 3D-printed proximal ulnar endoprosthesis was employed to restore elbow function (Figure 1D) (Liang et al., 2022a).

For both proximal radial and distal ulnar endoprostheses, we consider 8 cm to be the minimal defect length suitable for GFRS reconstruction. This threshold is based on two main considerations: first, from a manufacturing perspective, 8 cm is the minimum length required for the design and fabrication of a stable and functional endoprosthesis; second, from a clinical standpoint, defects shorter than 8 cm generally do not compromise forearm stability in adult patients, as the intact interosseous membrane is sufficient to maintain the integrity of the dual-bone structure.

### 2.2 Finite element analysis

Finite element analysis (FEA) was conducted to assess the impact of GFRS endoprostheses on stress distribution in residual bones. The DICOM data from a CT scan of a normal adult forearm were imported into Mimics Research 19.0.0.347 (Materialise, Belgium). Volume reconstruction was carried out, and cortical bone data were extracted and exported to Geomagic Studio 2014 (Geomagic, United States) for local editing and surface smoothing. The resulting STL file was then processed in Simcenter Nastran 2206 (Siemens Digital Industries Software, United States) for the final FEA.

**FIGURE 2:**
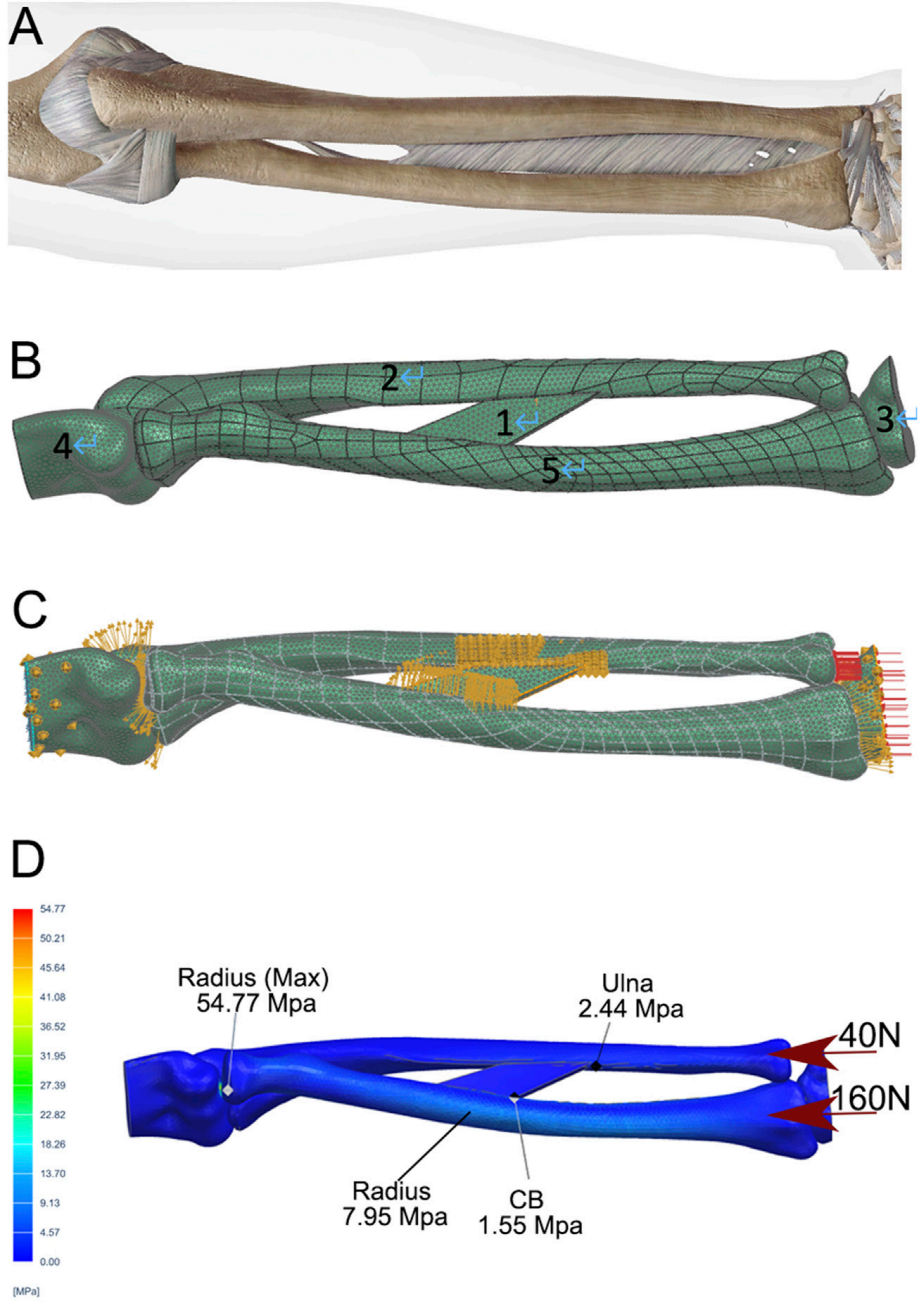
Development of a static FEA model of a normal forearm. **(A)** Depiction of the skeletal and ligamentous structures of the forearm. **(B)** DICOM data from a normal adult male, including the distal humerus (4), radius (5), ulna (2), and proximal carpal bones (3), were extracted, followed by volumetric reconstruction and surface smoothing. The interosseous membrane was modeled using the central band (CB, 1). A mesh was generated with a tetrahedron size of 2 mm. **(C)** The relationships between the elbow joint, humeroradial joint, wrist, and CB attachments were defined as direct contact and static bonding. **(D)** A compressive force of 200 N was applied to the distal radius and ulna, with a 4:1 distribution. Stress distribution was then calculated using the FEA method, with peak stresses shown for the ulna, radius, and CB.

The fundamental parameters for the FEA are summarized in Table 1, as previously reported (Ito et al., 2022; Kholinne et al., 2021; Andreani et al., 2020; Samiezadeh et al., 2019; Katsamanis and Raftopoulos, 1990). The forearm structure was simplified into five key components (Figures 2A,B): the intraosseous membrane (component 1), the ulna (component 2), the carpus (component 3), the distal humerus (component 4), and the radius (component 5). The intraosseous membrane was represented by the central band (CB), which originates from 60% of the radial length (measured from the radial styloid), extends distally and towards the ulna at a 20° angle, and inserts into the ulna at the junction of its middle and distal thirds (Masouros et al., 2020). The relationships among the joints (humeroulnar, humeroradial, radiocarpal, and ulnocarpal), and the attachment of the CB were defined as tied contact. A constant compressive force of 160 N was applied perpendicular to the distal radius articular surface, with 40 N to the distal ulna, simulating a total static loading of 200 N from the wrist.

**TABLE 1:** Parameters for finite element analysis.

Components	Young’s modulus (Mpa)	Poisson’s ratio	Thickness (mm)	Tetrahedral mesh (mm)
Cortical bone	16,200	0.36	2.3∼3	2
Central band	13.4	0.3	3.5	2
Ti6Al4V	110,000	0.3		
polyethylene	564	0.23		

**FIGURE 3:**
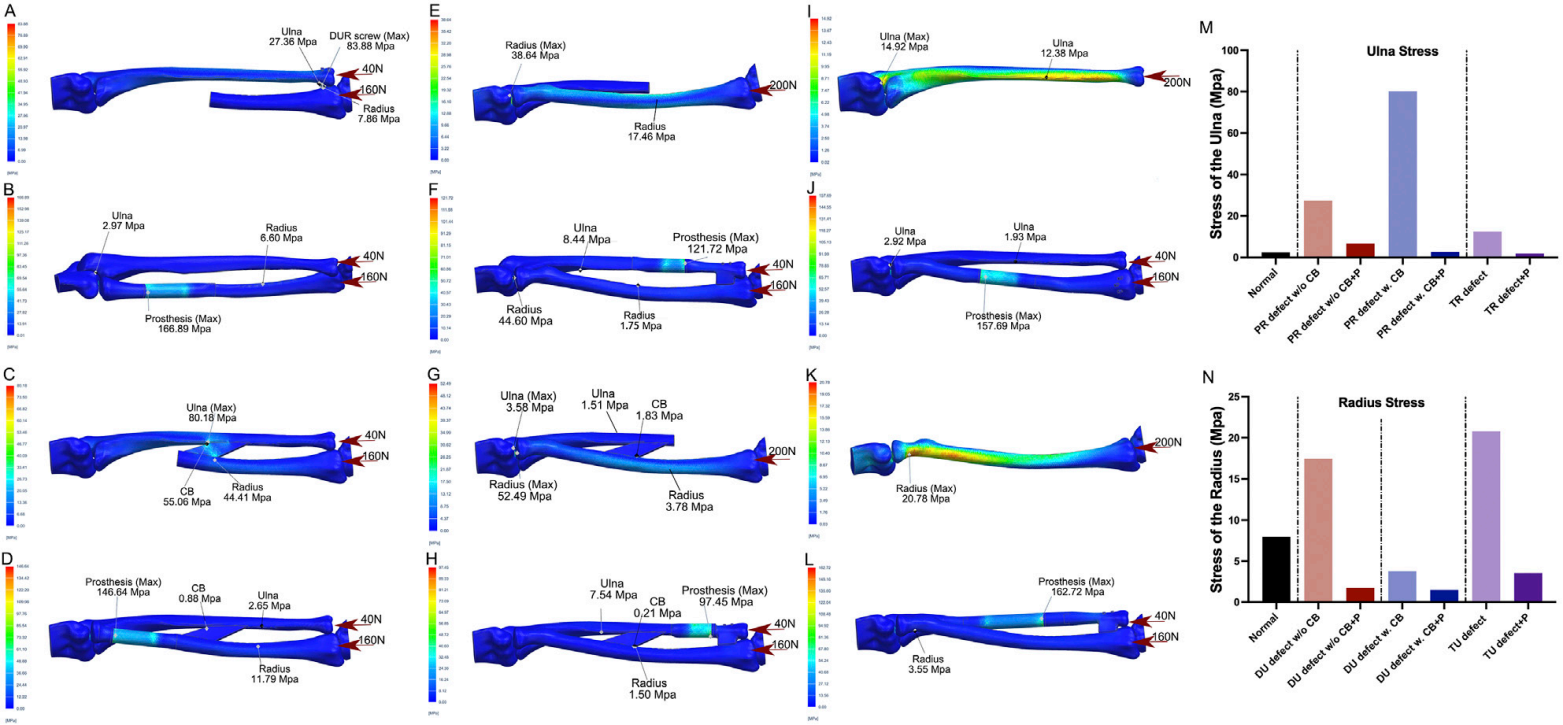
Illustrations of FEA results of various bone defects and their corresponding prosthetic reconstructions **(A,B)** A model of a proximal radial defect without the central band (CB) was simulated. In the case of reconstruction using distal ulnoradial fixation, high stress was observed at the distal ulna, distal radius, and screws when a 200 N compressive force, distributed 4:1 between the radius and ulna, was applied **(A)**. Reconstruction using a proximal radial endoprosthesis reduced the stress on the ulna and radius to levels similar to normal, though the prosthesis experienced relatively high stress at the junction of the telescoping structure **(B)**. **(C,D)** A proximal radial defect with the CB intact was modeled. Without reconstruction, high stress was concentrated at the CB and its attachment points on the ulna and radius under the same 200 N compressive force **(C)**. Reconstruction with a proximal radial endoprosthesis reduced the high stress around the CB to near-normal levels, but stress remained relatively high in the prosthesis at the junction of the telescoping structure **(D)**. **(E,F)** For a distal ulnar defect without the CB, leaving it unreconstructed resulted in increased stress in the proximal and diaphyseal regions of the radius under a 200 N load. Reconstruction using a distal ulnar endoprosthesis reduced the stress on the radius to near-normal levels, though the prosthesis bore relatively high stress at the junction of the telescoping structure. **(G,H)** In the case of a distal ulnar defect with the CB intact, the proximal radius experienced significant elevated stress under the same compressive force, while the diaphyseal radius experienced slightly decreased stress. When reconstructed with a distal ulnar endoprosthesis, the stress in both the ulna and radius decreased to even lower levels than normal, though relatively high stress persisted in the prosthesis at the junction of the telescoping structure. **(I,J)** A total radial defect was simulated, and without reconstruction, increased stress was seen in the ulna under the 200 N load. Reconstruction with a total radial endoprosthesis significantly reduced the stress on the ulna, but relatively high stress was observed in the prosthesis at the junction of the telescoping structure. **(K,L)** For a total ulnar defect, leaving it unreconstructed led to increased stress in the proximal radius under the 200 N compressive force. Reconstruction with a total ulnar endoprosthesis dramatically decreased the stress on the radius, although relatively high stress was still present in the prosthesis at the junction of the telescoping structure. **(M,N)** Bar charts illustrate the stress changes in the ulna **(M)** and radius **(N)** under various conditions, highlighting the impact of different reconstructions. (PR-proximal radius, w/o-without, CB-central band, w.-with, P-prosthesis, TR-total radius, DU-distal ulna, TU-total ulna).

FEA was performed under the following conditions: intact bones (Figures 2C,D); PR defect without CB (Figure 3A, two screws used for DURJ fixation); PR defect with CB (Figure 3C); DU defect without CB (Figure 3E); DU defect with CB (Figure 3G); TR defect (Figure 3I, 200 N load applied entirely to the ulna); TU defect (Figure 3K, 200 N load applied entirely to the radius); reconstruction with PR endoprosthesis (Figures 3B,D), DU endoprosthesis (Figures 3F,H), TR endoprosthesis (Figure 3J), and TU endoprosthesis (Figure 3L).

For endoprosthetic reconstruction models, the relationships between the intramedullary stem and residual bone, as well as the components fixed to adjacent bones by screws, were defined as tied contact, while the telescoping structure was defined as mobile.

### 2.3 *Ex vivo* installation of GFRS endoprostheses and testing of pronation/supination function

To simulate the installation process of GFRS endoprostheses and assess the pronation/supination function, we fabricated resin forearm models with different radius and ulna defects. As well as nylon prototypes of the GFRS endoprostheses, all using 3D-printing technology.

Installation of the PR endoprosthesis involved the following steps.

(1) Reaming the distal canal and fix the intramedullary stem.
(2) Inserting the polyethylene sleeve and the proximal component.
(3) Reducing the radial head and reconstructing the soft-tissue attachments.

**FIGURE 4:**
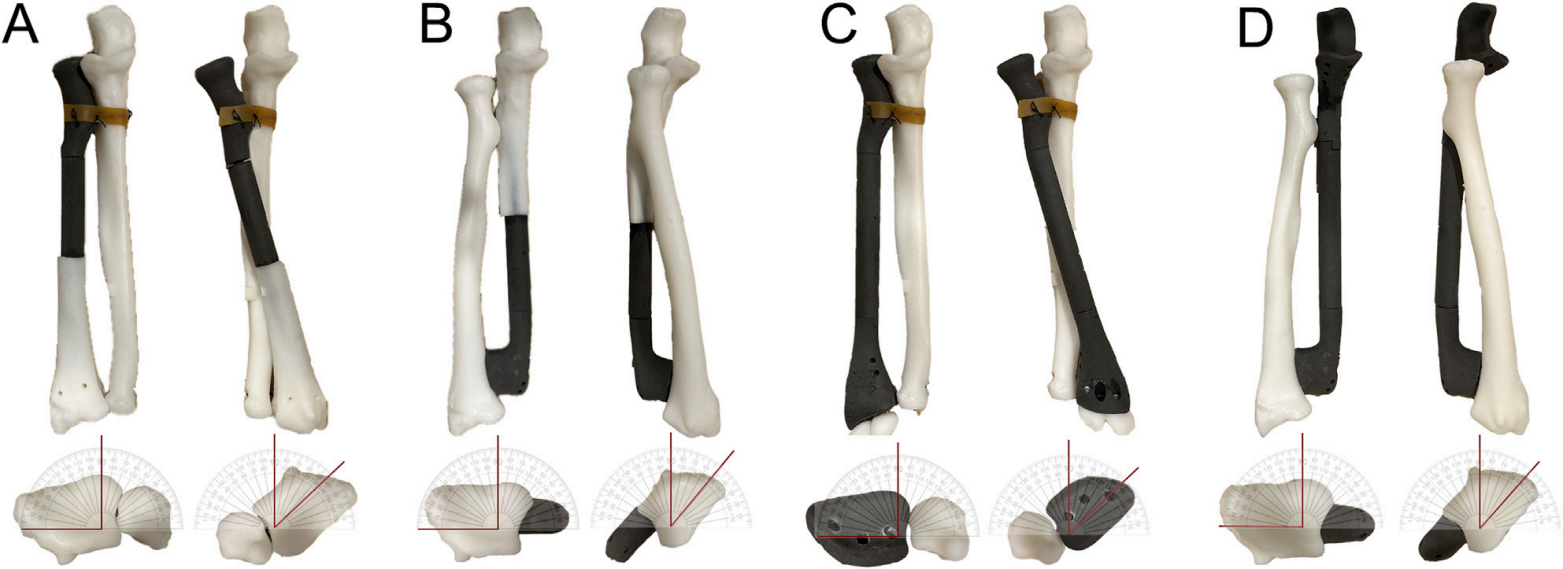
*Ex vivo* testing of the rotational function of GFRS prostheses. **(A)** A resin model simulating a proximal radial defect with reconstruction using a proximal radial endoprosthesis was created. The annular ligament reconstruction was mimicked by securing the prosthesis to the proximal ulna with a rubber band. The model demonstrated a maximum of 90° supination and 45° pronation without significant tension. **(B)** A resin model simulating a distal ulnar defect with reconstruction using a distal ulnar endoprosthesis was created. The model achieved a maximum of 90° supination and 40° pronation without noticeable tension. **(C)** A resin model simulating a total radial defect with reconstruction using a total radial endoprosthesis was created. The annular ligament reconstruction was simulated similarly by attaching the prosthesis to the proximal ulna with a rubber band. The model demonstrated a maximum of 90° supination and 45° pronation without significant tension. **(D)** A resin model simulating a total ulnar defect with reconstruction using a total ulnar endoprosthesis was created. The model demonstrated a maximum of 90° supination and 40° pronation without noticeable tension.

For the DU endoprosthesis, the stem was fixed first, followed by assembly of the telescoping structure and fixation of the distal component. Installation of the TR and TU endoprostheses started with the fixation of the distal components and concluded with soft tissue reconstruction at the proximal sites. Following installation, the maximum achievable pronation and supination of the endoprostheses were tested (Figure 4).

### 2.4 Preliminary clinical application

Between 2021 and 2022, three patients with bone-derived sarcomas were treated at our center: two with total ulna resection (one osteosarcoma and one Ewing sarcoma), and one with osteosarcoma involving the proximal 70% of the radius. The GFRS endoprostheses were applied as a salvage method as there were no other feasible alternative options for reconstruction. The study was approved by the institutional review board of Peking University People’s Hospital (2024PHB432-001), and written informed consent was obtained from the guardians of each patient.

During the final cycle of neoadjuvant chemotherapy, all patients underwent CT and MRI scans of the forearm, from which DICOM data were obtained. Once the resection plan was confirmed, prosthesis design began based on the principles described earlier, with particular attention to components requiring high contour compatibility with the host bones, such as the distal component of the TR endoprosthesis. Screw trajectories direction and length were customized according to the DICOM data.

The metal components of the endoprostheses were fabricated using electron beam melting (EBM) 3D-printing technology with Ti6Al4V powder, while the polyethylene components were produced via conventional methods (Chunli, Beijing, China). The DURJ interface was porous with a proper pore size (500 μm) and a porosity rate (60%) that facilitated bone ingrowth. Intraoperative data were collected, and adjuvant chemotherapy was initiated 2 weeks post-surgery. Post-operative follow-up was conducted as per standard protocols—every 3 months for the first 2 years, every 4 months in the third year, every 6 months in the fourth and fifth years, and annually thereafter. Oncological and functional outcomes were assessed at each follow-up visit.

## 3 Results

### 3.1 Improved stress distribution in residual bones with GFRS endoprostheses

For a PR defect without preservation of the intraosseous membrane (IOM), the finite element analysis (FEA) revealed a peak stress of 83.88 MPa at the radioulnar screws and increased stress in the ulna (27.36 MPa vs. 2.44 MPa in normal conditions). When the IOM was preserved, stress levels increased in both the IOM and the diaphyseal ulna (55.06 MPa and 80.18 MPa, respectively) compared to normal status (1.55 MPa and 2.44 MPa). Reconstruction using the PR endoprosthesis alleviated stress in the ulna and IOM (<3 MPa), though peak stresses were observed within the implant itself (166.89 MPa and 146.64 MPa) (Figures 3A–D,M).

For a DU defect without IOM preservation, a moderate increase in stress was observed in the diaphyseal radius (17.46 MPa vs. 7.95 MPa in normal conditions), with the peak stress located at the humeroradial articular surface (38.64 MPa). When the IOM was preserved, the stress distribution in the diaphyseal and proximal radius remained similar to normal conditions (3.78 MPa vs. 7.95 MPa, and 52.49 Mpa vs. 54.77 Mpa, respectively). Reconstruction with the DU endoprosthesis significantly reduced stress in the radius (<2 MPa), but a peak stress was noted within the implant (121.72 MPa and 97.45 MPa) (Figures 3E–H,N).

For the TR defect, stress in the diaphyseal ulna increased significantly compared to normal status (12.38 MPa vs. 2.44 MPa). This stress was reduced following reconstruction with the TR endoprosthesis (<2 MPa). Similar findings were observed in the TU defect (Figures 3I–N).

### 3.2 Satisfactory rotational function of GFRS endoprostheses in an in vitro model

The mobility of the GFRS endoprostheses in terms of pronation and supination was assessed using a resin model. The maximum pronation achieved with the PR, DU, TR, and TU endoprostheses was 45°, 40°, 45°, and 40°, respectively. For all four endoprostheses, the maximum supination reached 90° (Figure 4).

### 3.3 Safety and efficacy of GFRS endoprostheses in preliminary clinical application

The GFRS endoprostheses were used for reconstruction in two cases of TU defect and one case of PR defect, with no surgery-related complications observed.

**FIGURE 5:**
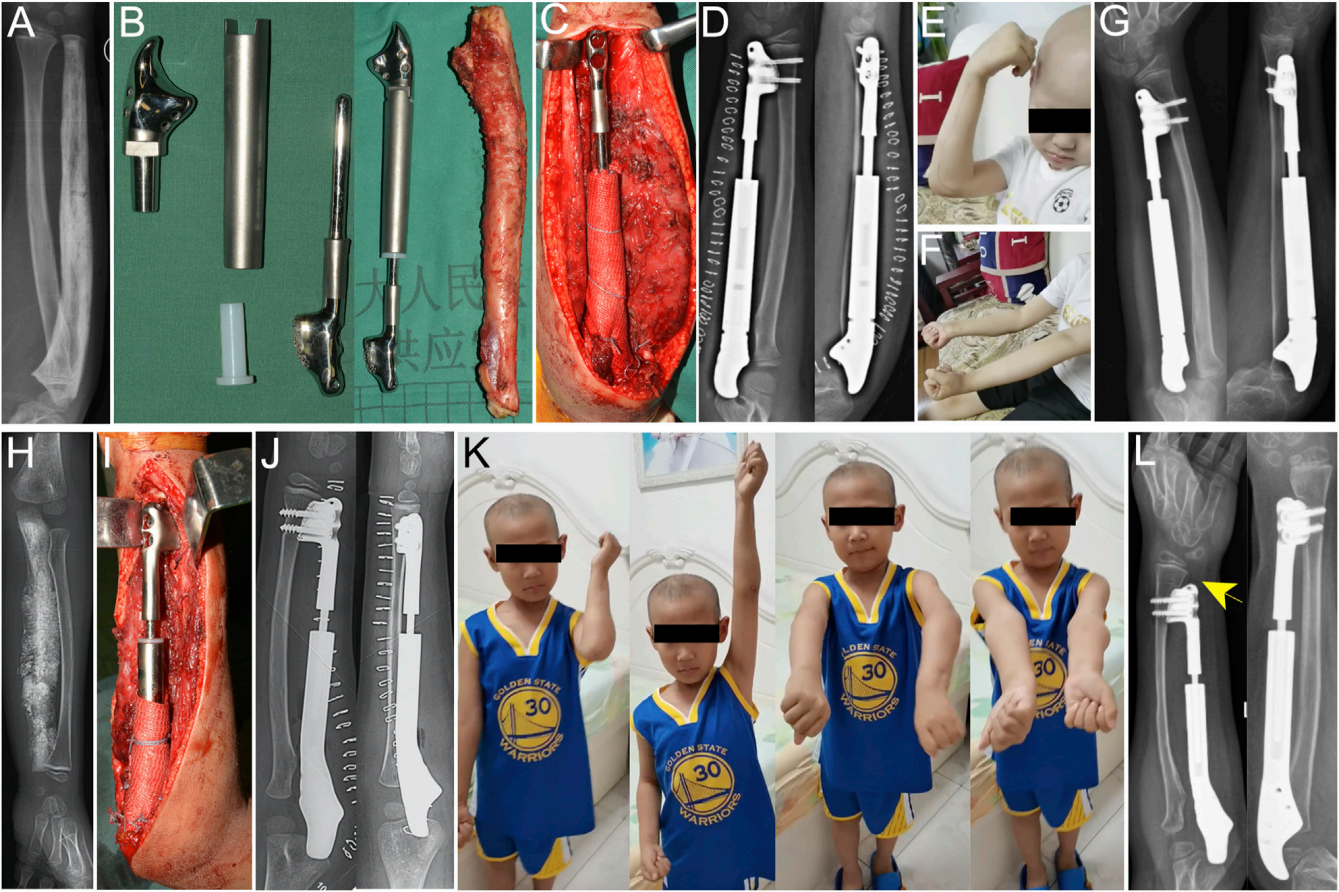
Application of the total ulnar endoprosthesis in Case 1 **(A–G)** and Case 2 **(H–L)**. **(A)** Post-neoadjuvant chemotherapy X-ray of a 7-year-old boy with Ewing sarcoma of the right ulna. **(B)** The endoprosthesis consisted of four parts that could be assembled to form a telescoping structure. **(C)** Intraoperative photo after the installation of the endoprosthesis. A LARS ligament was used to repair the capsule and the triceps insertion. **(D)** A postoperative X-ray showed the endoprosthesis in a good position. **(E)** The patient achieved a range of motion (ROM) of 30°–120°. **(G)** A follow-up X-ray after 42 months revealed a slight ulnar inclination of the wrist. **(H)** X-ray image of a patient with osteosarcoma in the left ulna following neoadjuvant chemotherapy. **(I)** An intraoperative photo after implantation of the endoprosthesis. **(J)** Postoperative X-ray showing the positioning of the implant. **(K)** The patient regained nearly full function of the upper limb. **(L)** A follow-up after 35 months indicated a discrepancy between the ulna and radius (yellow arrow).

Case 1 A 7-year-old boy diagnosed with stage IIB Ewing sarcoma in the right ulna underwent neoadjuvant chemotherapy, followed by a TU resection with a wide margin and reconstruction with a GFRS endoprosthesis. The operation lasted 120 min, with intraoperative blood loss of 30 mL. Intraoperative testing showed full mobility of the forearm (Supplementary Video S1). At the 42-month follow-up (cut-off date: 1 September 2024), the patient remained disease-free, with an MSTS-93 score of 28 and an MEPS score of 95. The elbow range of motion (ROM) was 30°–120°, with forearm pronation and supination were 30° and 80°, respectively (Figures 5A–G). The wrist joint ROM was comparable to the contralateral side.

Case 2 An 8-year-old boy diagnosed with stage IIB osteosarcoma in the left ulna. Following neoadjuvant chemotherapy, he underwent TU resection with a wide margin and reconstruction. The operation lasted 180 min, with intraoperative blood loss of 100 mL. At the 35-month follow-up (cut-off date: 1 September 2024), the patient was disease-free, with an MSTS-93 score of 28 and an MEPS score of 100. The elbow ROM was 0°–135°, with forearm pronation and supination of 60° and 90°, respectively (Figures 5H–L). He also showed similar ROM of the wrist joint compared with the healthy side.

**FIGURE 6:**
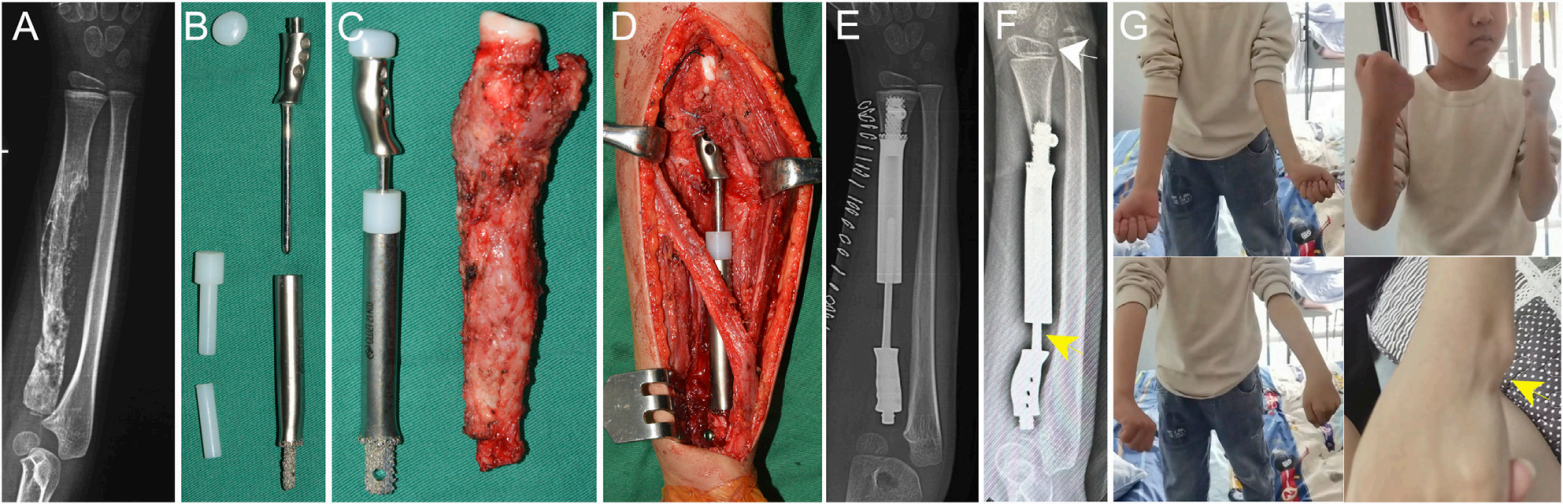
Application of the proximal radial endoprosthesis in Case 3. **(A)** X-ray image of a patient with osteosarcoma in the proximal radius following neoadjuvant chemotherapy. The estimated residual radius after tumor resection was too short to accommodate a regular stem. **(B)** The components of the proximal radial endoprosthesis. The endoprosthesis adopted a 3D-printed short stem with an interlocking screw to achieve fixation. **(C)** The endoprosthesis alongside the excised specimen. **(D)** Intraoperative photo taken after the installation of the endoprosthesis. The telescoping structure was used to adjust the length of the endoprosthesis. **(E)** Postoperative X-ray demonstrating the positioning of the implant. **(F)** A follow-up after 32 months revealed radial inclination of the wrist (white arrow). A proximal migration of the prosthesis was observed (yellow arrow). **(G)** The patient regained nearly full function of the upper limb. Protrusion of the distal ulna was seen due to the proximal migration of the prosthesis (yellow arrow).

Case 3 A 5-year-old boy diagnosed with stage IIB osteosarcoma in the right radius. After neoadjuvant chemotherapy, he underwent resection of the PR with a wide margin, leaving less than 2 cm of the radius intact. The operation lasted 100 min, with intraoperative blood loss of 25 mL. Intraoperative testing showed full mobility of the forearm (Supplementary Video S2). At the 32-month follow-up (cut-off date: 1 September 2024), the patient was disease-free, with an MSTS-93 score of 29 and an MEPS score of 100. The elbow ROM was 0°–135°, with forearm pronation and supination were 70° and 90°, respectively (Figure 6). The ROM of the wrist joint was comparable between the affected and contralateral limbs.

## 4 Discussion

Reconstruction methods for defects of the proximal radius (PR), distal ulna (DU), total ulna (TU), and total radius (TR), as well as their applications in these rare scenarios, remain uncertain. In this proof-of-concept study, we demonstrated that these four types of bone defects led to stress concentration in the residual structures, which could be alleviated by the corresponding GFRS endoprostheses. We also provided preliminary evidence of the rotational function of each GFRS endoprosthesis in an *ex vivo* model. Finally, we confirmed the safety and efficacy of the TU and PR endoprostheses in three clinical cases with mid-term follow-up.

### 4.1 Mechanical support provided by GFRS endoprostheses

Currently, no literature has assessed the changes in load distribution after the resection of PR, DU, TU, or TR bone tumors. Based on our FEA results, we found that a defect in the PR can lead to significantly increased stress on the ulna and/or the intraosseous membrane (IOM), a pattern not observed in DU defects. These findings align with the basic biomechanical concept that 80% of carpal loading is transferred through the radius, with 60% passing through the humeroradial joint (Masouros et al., 2020).

If a PR defect is left unreconstructed, any connection between the remaining radius and ulna, such as the IOM or ulnoradial fixing screws (either distal or proximal), becomes a focal point of stress concentration (Figures 3A,C; Supplementary Figure S1). This increases the risk of screw loosening, ulnar fractures, and subsequent proximal migration of the radius. Reconstruction with the PR endoprosthesis restored direct mechanical support to the radial column, significantly reducing the load on the ulna (Figures 3B,D,M), thereby decreasing the risk of ulnar fracture and proximal radial migration. A similar principle applies to TR defects, where surgical centralization of the carpus on the distal end of the ulna was assumed as a non-prosthetic reconstruction method (Figures 3I,J,M) (Sestero et al., 2006).

The need for DU reconstruction is minimal regarding mechanical support, especially when the IOM is preserved (Figures E–H,N). This is consistent with previous findings in the treatment of distal ulnoradial joint (DURJ) disorders (Minami et al., 2010; Papanastassiou et al., 2019). Conversely, reconstruction a TU defect is crucial, as it not only reduces stress concentration on the radius but also restores the functional structure of the elbow joint (Figures 3K,L,N).

In conclusion, defects in the PR, DU, TU, or TR may lead to abnormal axial loading of the remaining bones, which can be effectively addressed using GFRS endoprostheses. Additionally, the FEA showed high stress concentration at the junction of the telescoping structure, although these stress levels were well below the Young’s modulus of Ti6Al4V. The long-term implications of this finding remain unclear.

### 4.2 Rotational function of GFRS endoprostheses

Forearm pronation and supination involve the rotation of the radiocarpal unit around a rotationally fixed and stable ulna (Kleinman, 2007). In addition to the action of muscles like the pronator teres, pronator quadratus, supinator, brachioradialis, and biceps, successful pronation and supination require a “stable” yet “mobile” DURJ and PURJ (Kleinman, 2007). Achieving this delicate balance has been challenging with conventional methods, such as reattaching ligaments to a single-bloc prosthesis, as scar tissue formation during stabilization tends to limit rotational range.

To address this challenge, we implemented a telescoping structure, assigning stability and mobility to separate components (Figures 1, 4). The proximal part of the PR and TR endoprostheses and the distal part of the DU and TU endoprostheses, was designed to provide stability of the DURJ and PURJ, while the telescoping structure enabled rotational movement. To evaluate the rotatory function, we tested a simplified resin model. The model demonstrated full supination capability (100%) and approximately 50% of pronation. The limitation in pronation might have been due to the limitations of the testing method and potential impingement of the telescoping components during rotation.

Additionally, our preliminary clinical study showed improved pronation and supination range *in vivo* after installation of the PR and TU endoprostheses compared with the *ex vivo* results (Supplementary Videos S1,S2). Overall, our findings suggest that the telescoping structure of the GFRS endoprostheses effectively and safely restores pronosupination function.

### 4.3 Preliminary clinical results of GFRS endoprostheses

Given the rarity of bone malignancies in the forearm, the GFRS endoprostheses have been applied in only two cases of TU defects and one case of PR defect. The results indicated that the TU endoprostheses were successful in restoring elbow flexion, extension, as well as forearm pronation and supination. However, we observed that the range of motion (ROM) in Case 1 was more restricted compared to Case 2, likely due to the extended immobilization period in Case 1 (2 weeks) versus Case 2 (3 days). Both patients were skeletally immature, and we noted that the distal radius outgrew the endoprosthesis over time. Despite this discrepancy, no negative effects on wrist appearance or function were observed (Figure 5).

In the case of the PR defect, satisfactory function of the elbow, forearm, and wrist was also achieved. However, significant subsidence of the distal radius was noted, leading to slightly prominence of the distal ulna (Figure 6G). This issue aligns with the FEA results (Figure 3B), which showed high stress concentration at the junction of the telescoping structure. During the initial surgery, we extended the endoprosthesis using the telescoping structure to compensate for an unexpected length deficit, leaving no direct contact between the junction components. This likely caused the distal part to slide backward under consistent wrist pressure (Figures 6E,F). However, the patient was satisfied with current functional status and appearance, and refused further revision surgeries by last follow-up. For future improvements to the PR endoprosthesis, we suggested that the tip of the smooth rod and the bottom of the sleeve should maintain contact with each other to prevent longitudinal sliding of the telescoping structure.

### 4.4 Limitations

This study had several limitations. First, the FEA calculated only a single static stress distribution, which does not fully represent the conditions during rotational movement. Second, while we included FEA for scenarios with and without preservation of the IOM, we did not explore the full value of the IOM in depth. Third, the follow-up duration in this study was not sufficient to determine whether the stress concentrations observed in the telescoping structure will eventually result in mechanical failure, nor to evaluate the compatibility of these prostheses with skeletal growth in pediatric patients. Fourth, the impact of additional soft-tissue reconstruction remains unclear, as we only reconstructed the soft-tissue attachments around the elbow in the three cases. However, the mid-term outcomes did show that such reconstruction can lead to satisfactory functional results. Fifth, in skeletally immature patients, reconstruction with a total radial prosthesis may lead to significant length discrepancies between the radius and ulna over time, potentially resulting in wrist deformities. At present, we lack sufficient knowledge and clinical experience to effectively address this challenge. Finally, the small number of clinical cases with heterogeneity and the lack of comparison group in this study limit the strength of our conclusions, which is an inevitable consequence of the rarity of such cases.

## 5 Conclusion

Although forearm malignancies are rare, the resulting bone defects in the radius and/or ulna require appropriate reconstruction to restore mechanical support and facilitate basic movements. In this proof-of-concept study, we demonstrated that the GFRS endoprostheses fulfill theoretical reconstruction requirements, exhibit a favorable safety profile, and produce satisfactory functional outcomes in a preliminary cohort with mid-term follow-up.

## Data Availability

The original contributions presented in the study are included in the article/Supplementary Material, further inquiries can be directed to the corresponding authors.

## Supplementary material

SUPPLEMENTARY FIGURE S1 FEA analysis and clinical observation of a case of proximal radial defect and reconstruction. **(A)** A model of a proximal radial defect without the central band (CB) with reconstruction using proximal ulnoradial fixation was simulated. High stress was observed at the distal ulna, distal radius, and screws when a 200 N compressive force, distributed 4:1 between the radius and ulna, was applied. **(B–E)** A case of osteosarcoma in the proximal radius **(B)** was treated by *en bloc* resection and reconstruction by screw fixation and grafting at the proximal end of radius **(C)**. Fracture of the ulna was observed 9 months after surgery **(D)**, and was treated by internal fixation **(E)**.
