# Supplementary figures and images for: Development of a global forearm reconstruction system for post-tumor resection defects of the radius or ulna: a proof-of-concept study
Source: Front Bioeng Biotechnol. 2025 Jun 11;13:1547652. doi: 10.3389/fbioe.2025.1547652 (PMC12187641; doi:10.3389/fbioe.2025.1547652)

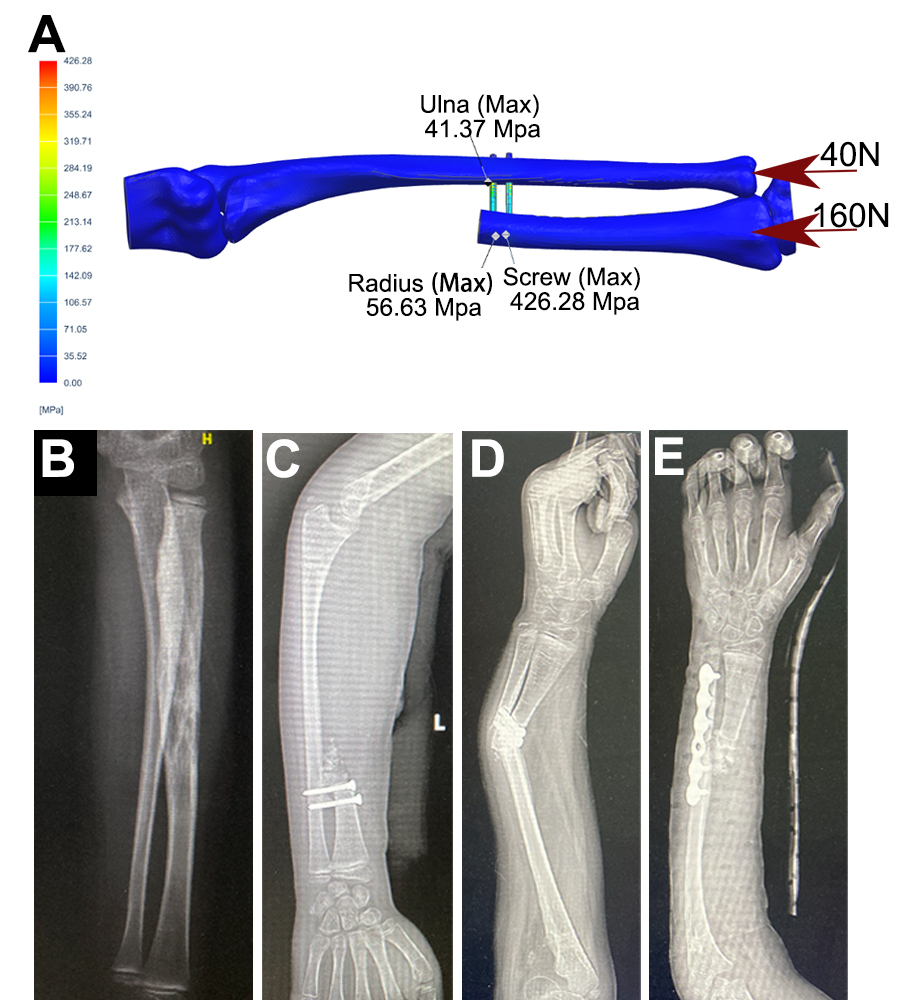

Supplement: Supplementary file 2 [file Image1.jpeg]
